# Effect of acute predation with bacteriophage on intermicrobial aggression by *Pseudomonas aeruginosa*

**DOI:** 10.1371/journal.pone.0179659

**Published:** 2017-06-16

**Authors:** Patrick R. Secor, Gabriele Sass, Hasan Nazik, David A. Stevens

**Affiliations:** 1 Department of Microbiology, University of Washington, Seattle, Washington, United States of America; 2 California Institute for Medical Research, San Jose, CA, United States of America; 3 Division of Infectious Diseases and Geographic Medicine, Department of Medicine, Stanford University, Stanford, CA, United States of America; 4 Department of Medical Microbiology, Faculty of Medicine, Istanbul University, Istanbul, Turkey; Laurentian, CANADA

## Abstract

In persons with structural lung disease, particularly those with cystic fibrosis (CF), chronic airway infections cause progressive loss of lung function. CF airways can be colonized by a variety of microorganisms; the most frequently encountered bacterial and fungal pathogens are *Pseudomonas aeruginosa* and *Aspergillus fumigatus*, respectively. Co-infection with *P*. *aeruginosa* and *A*. *fumigatus* often results in a more rapid loss of lung function, indicating that interactions between these pathogens affect infection pathogenesis. There has been renewed interest in the use of viruses (bacteriophage, mycoviruses) as alternatives to antibiotics to treat these infections. In previous work, we found that filamentous Pf bacteriophage produced by *P*. *aeruginosa* directly inhibited the metabolic activity of *A*. *fumigatus* by binding to and sequestering iron. In the current study, we further examined how filamentous Pf bacteriophage affected interactions between *P*. *aeruginosa* and *A*. *fumigatus*. Here, we report that the antifungal properties of supernatants collected from *P*. *aeruginosa* cultures infected with Pf bacteriophage were substantially less inhibitory towards *A*. *fumigatus* biofilms. In particular, we found that acute infection of *P*. *aeruginosa* by Pf bacteriophage inhibited the production of the virulence factor pyoverdine. Our results raise the possibility that the reduced production of antimicrobials by *P*. *aeruginosa* infected by Pf bacteriophage may promote conditions in CF airways that allow co-infection with *A*. *fumigatus* to occur, exacerbating disease severity. Our results also highlight the importance of considering how the use of bacteriophage as therapeutic agents could affect the behavior and composition of polymicrobial communities colonizing sites of chronic infection.

## Introduction

The airways of people with cystic fibrosis (CF) are persistently colonized by various opportunistic pathogens, the most prevalent being *Pseudomonas aeruginosa* (Pa) [1] and *Aspergillus fumigatus* (Af) [2]. Compared to infection with either Af or Pa, concurrent infection with both pathogens is correlated with a more rapid decline in lung function [3], indicating that *in vivo* interactions between Af and Pa affects infection pathogenesis. However, interactions between Af and Pa are complex and incompletely understood. Recent work indicates that Af can promote virulence factor production by Pa [4]. Pa is also capable of producing a variety of antimicrobial compounds [5,6], especially during growth as a biofilm [7].

When bacteria and fungi form biofilms, they become inherently tolerant to antibiotics [8]. With the increasing prevalence of antibiotic resistant microorganisms, alternative strategies for controlling microbial infections have become a priority. One such strategy involves the use of viruses that infect and kill bacteria or fungi. Bacteriophage—viruses that infect bacteria—have been used to successfully treat Pa infections in mice [9] and human clinical trials utilizing bacteriophage therapy are currently being conducted [10]. However, some bacteriophage that infect Pa can promote bacterial phenotypes associated with infection. For example, when grown as a biofilm, Pa produces abundant filamentous Pf bacteriophage (Pf phage)—often greater than 10^10^ plaque forming units/ml [11,12,13]. The accumulation of Pf phage in the extracellular matrix of Pa biofilms enhances biofilm formation and promotes desiccation survival and antibiotic tolerance [11]. Pf phage are also capable of physically trapping bacteria in the lung, producing a non-invasive infection phenotype [14], a hallmark of most chronic infections.

In previous work, we found that Pf phage directly inhibited Af biofilms by chelating iron, denying Af this critical resource [15]. Here, we further investigated the interplay between Pf phage, Pa, and Af. We found that infection of Pa with Pf phage lowered the antifungal activity of Pa supernatants against Af biofilms and reduced the production of the virulence factor and siderophore pyoverdine. These findings are important because Pf phage are prevalent amongst clinical Pa CF isolates [16]. Thus, determining how Pf phage affect intermicrobial interactions will contribute to the knowledge base required for the development of new therapeutic strategies, and understanding current therapeutic approaches aimed at treating CF patients infected by both Pa and Af and/or other microbes.

## Results

**Fig 1:**
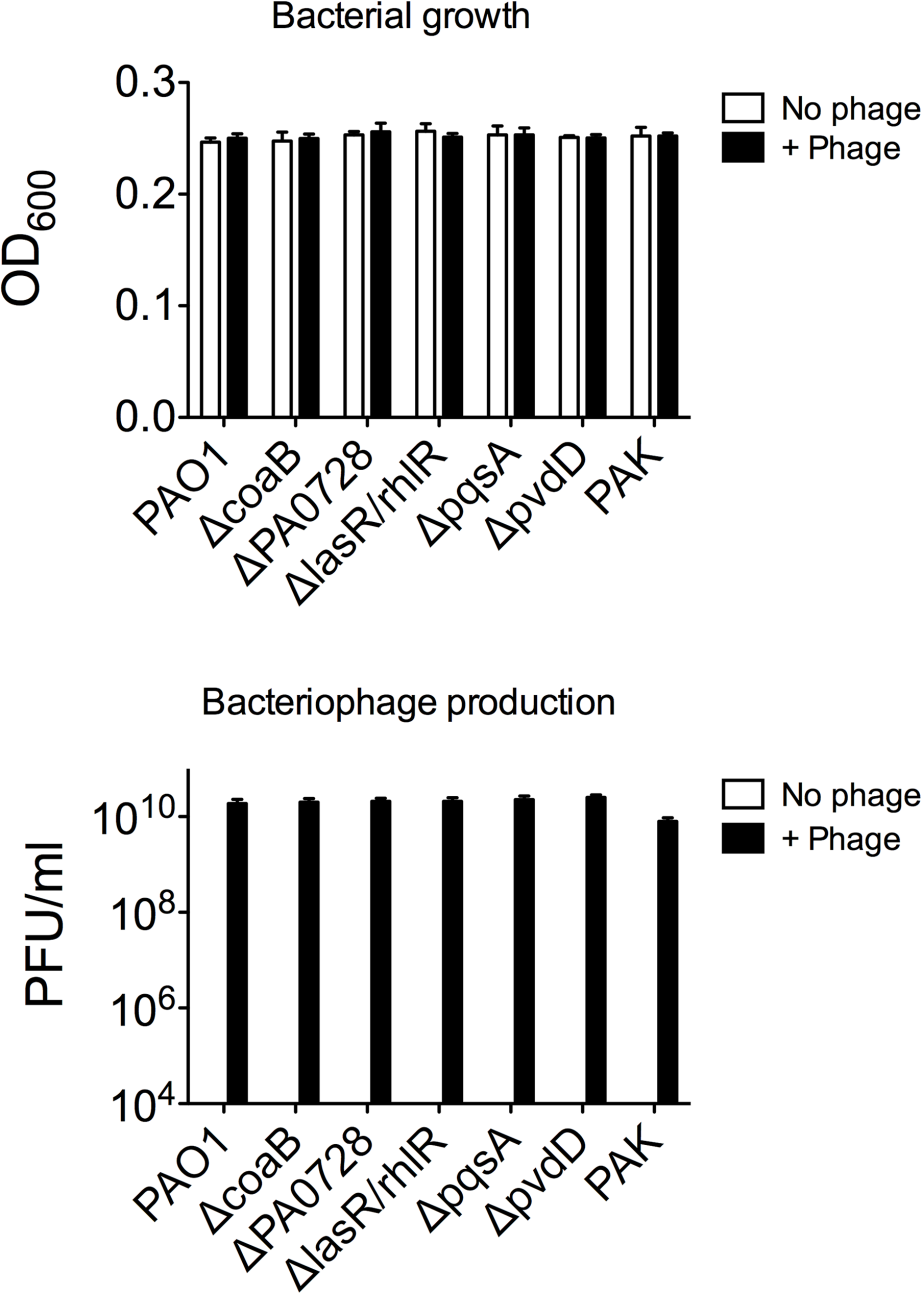
Enumeration of bacteria and Pf phage in *Pseudomonas aeruginosa* (Pa) supernatants. (A) After overnight growth, bacterial density was estimated by absorbance (OD_600_). (B) Pf phage in filtered supernatants collected from overnight cultures of the indicated *P*. *aeruginosa* strains were measured by enumerating plaque-forming units (PFU/ml) as described [14]. In all non-infected cultures, Pf phage levels were below the limit of detection of 2000 PFU/ml. N = 6 +/- SD mean.

**Fig 2:**
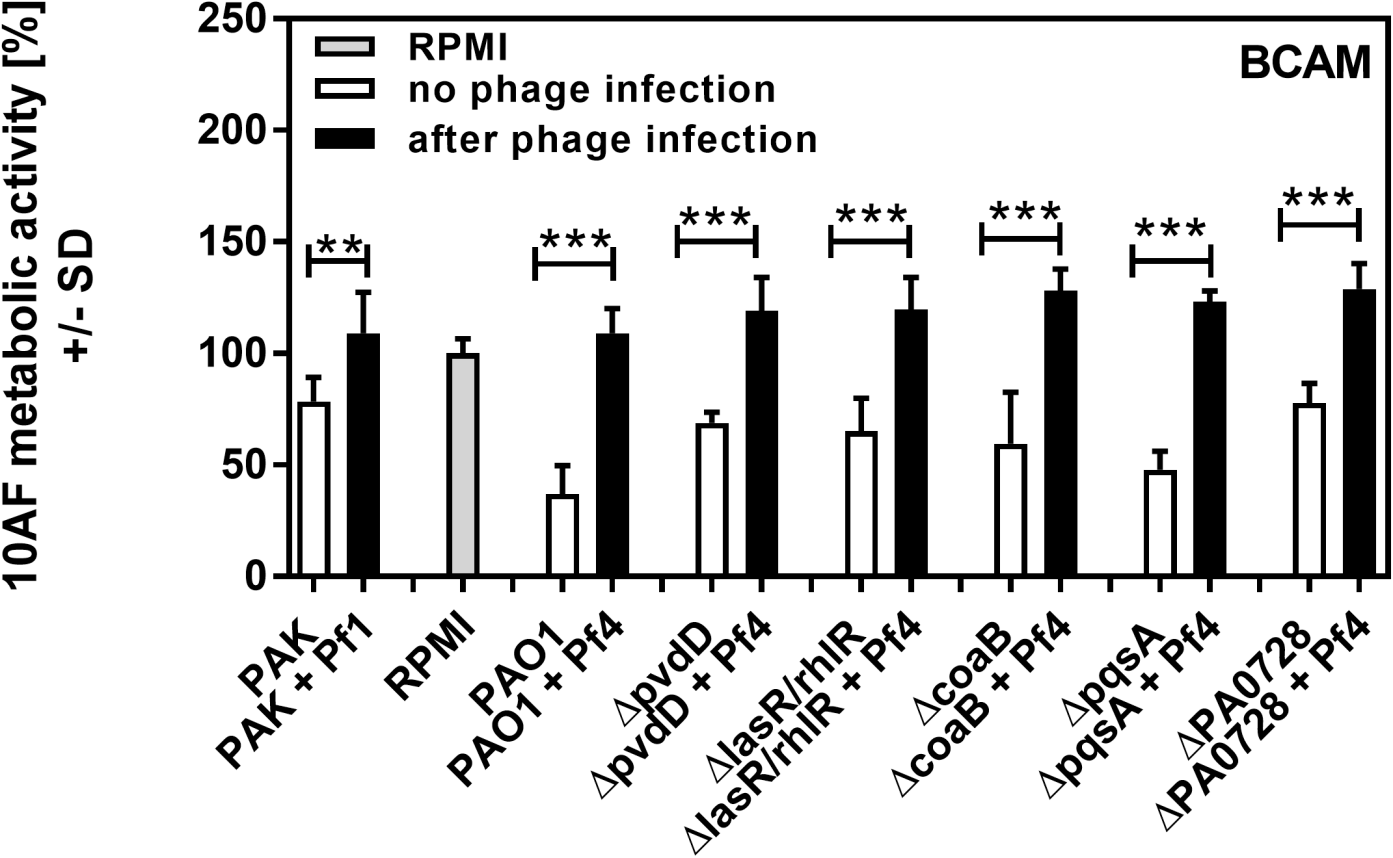
Effect of phage infection on formation of Af biofilm, as studied in an agar assay (BCAM). Black bars show inhibition of Af biofilm by planktonic supernatants of Pa after acute infection with phage, white bars show inhibition of Af biofilm by planktonic supernatants of the same strains of Pa cultured in the absence of phage. Grey bar shows negative control (RPMI medium only). Horizontal asterisks compare the bars at the ends of the horizontal brackets; two and three asterisks represent differences of p ≤ 0.01, p ≤ 0.001, respectively, comparing each Pa isolate uninfected vs. after phage infection. Comparison of all vs RPMI (the Af control, grey bar): PAO1+Pf4 or PAK+Pf1 p>0.05. *ΔpvdD*+Pf4 or *ΔlasR/rhlR*+Pf4: p ≤ 0.01. All other bars: p≤0.001. Comparison of PAO1 vs. its mutants: *ΔcoaB*: p≤0.05, *ΔlasR/rhlR*: p ≤ 0.01. *ΔpqsA*: p>0.05. All other bars: p≤0.001. Comparison PAO1 vs PAK: p≤0.001.

**Fig 3:**
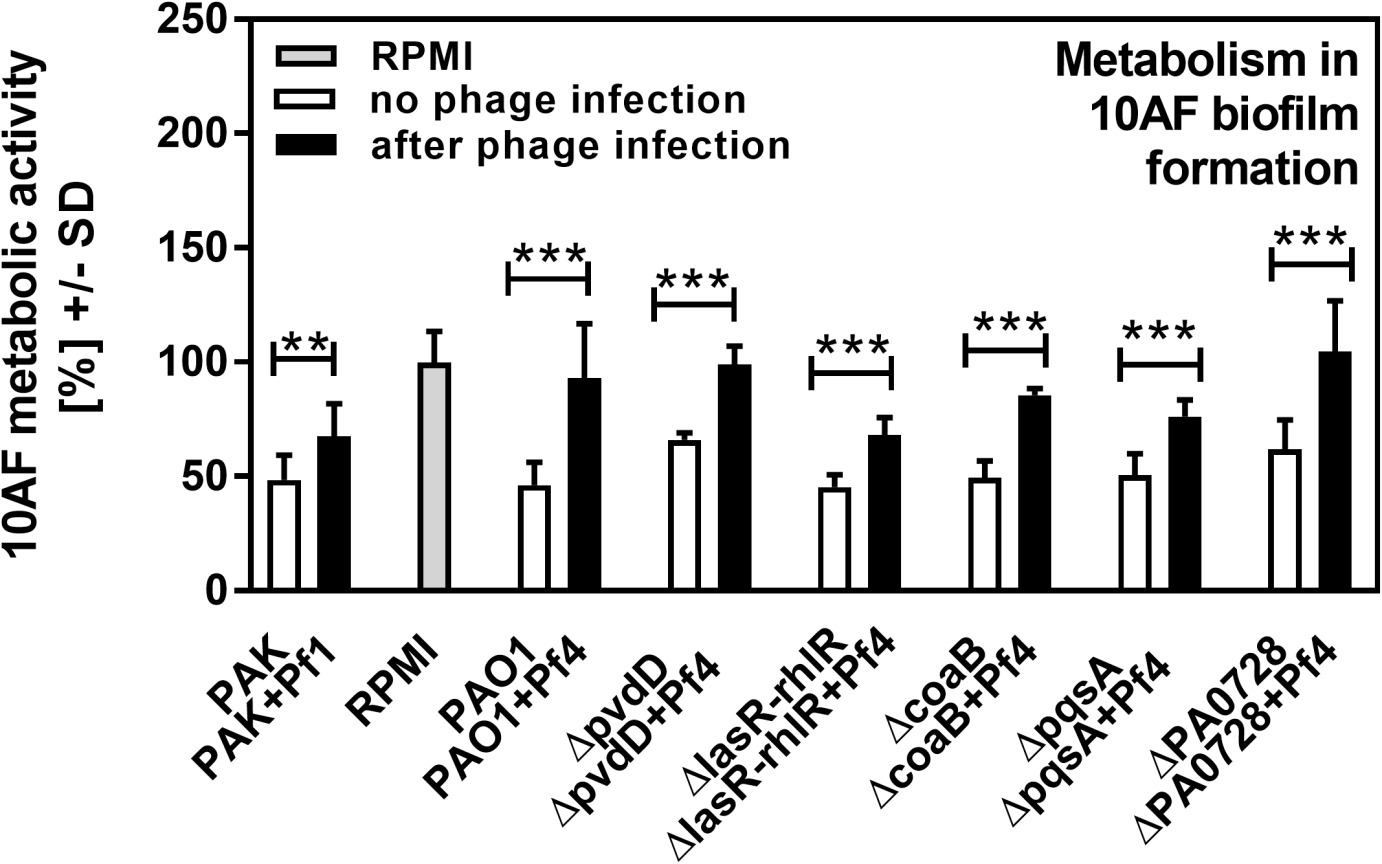
Effect of phage infection on formation of Af biofilm, as studied in liquid in wells. Black bars show inhibition of Af biofilm by planktonic supernatants of Pa after acute infection with phage, white bars show inhibition of Af biofilm by planktonic supernatants of the same strains of Pa cultured in the absence of phage. Grey bar shows negative control (RPMI medium only). Horizontal asterisks compare the bars at the ends of the horizontal brackets; two and three asterisks represent differences of p ≤ 0.01, p ≤ 0.001, respectively, comparing each Pa isolate uninfected vs. after phage infection. Comparison of all vs RPMI (the Af control, grey bar): *ΔcoaB+* Pf4: p ≤ 0.01; PAO1+Pf4, *ΔpvdD*+Pf4 and *ΔPAO728 +* Pf4: p>0.05. All other bars: p≤0.001. Comparison PAO1 vs. its mutants: *ΔpvdD* and *ΔPAO728*: p≤0.001. All other bars: p>0.05. Comparison PAO1 vs PAK: p≤0.001.

**Fig 4:**
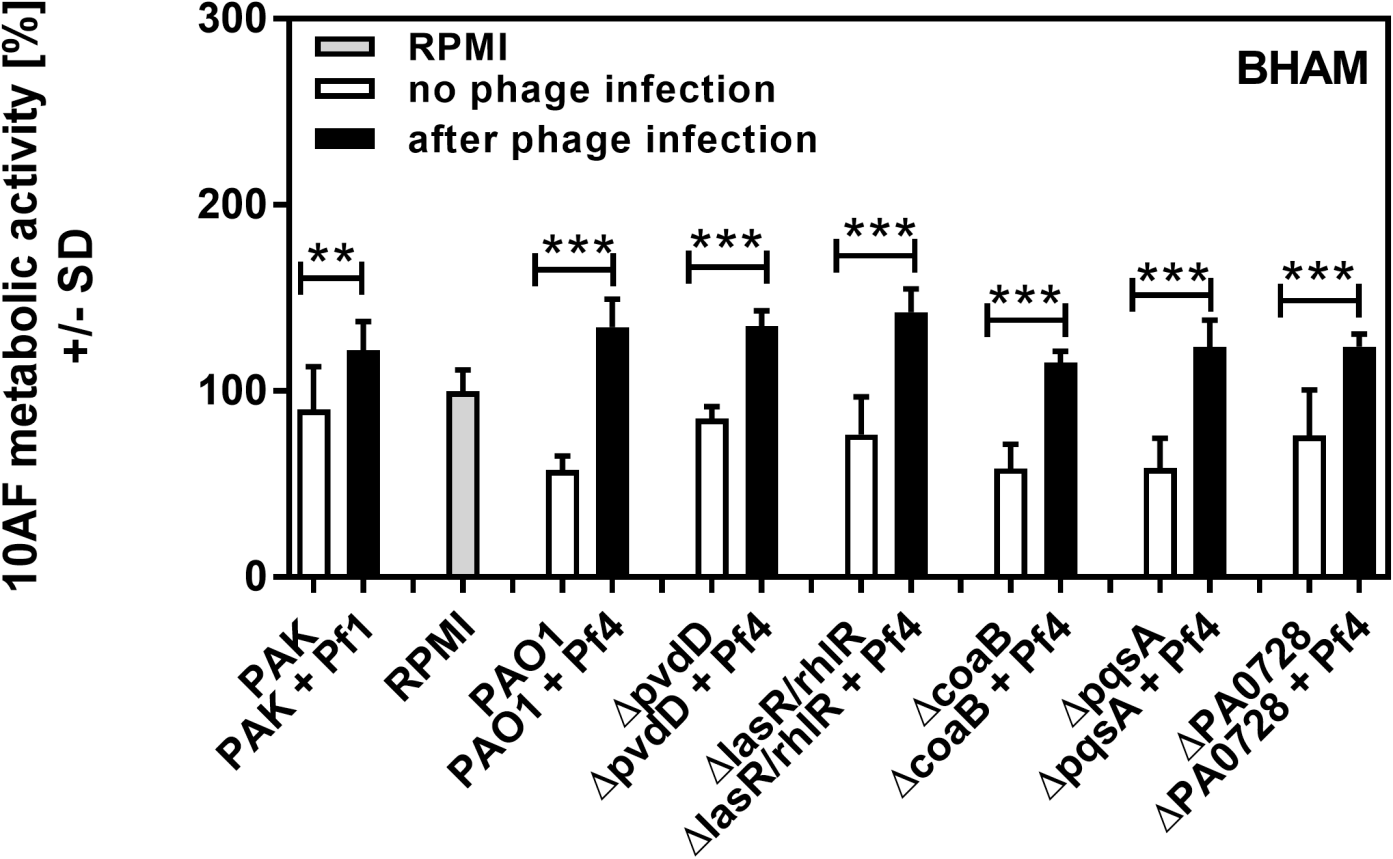
Effect of phage infection on preformed Af biofilm, as studied in an agar assay (BHAM). Black bars show inhibition of Af biofilm by planktonic supernatants of Pa after acute infection with phage, white bars show inhibition of Af biofilm by planktonic supernatants of the same strains of Pa cultured in the absence of phage. Grey bar shows negative control (RPMI medium only). Horizontal asterisks compare the bars at the ends of the horizontal brackets; two and three asterisks represent differences of p ≤ 0.01, p ≤ 0.001, respectively, comparing each Pa isolate uninfected vs. after phage infection. Comparison of all vs RPMI (the Af control, grey bar): *ΔlasR/rhlR* or *ΔPA0728*: p≤0.05; *ΔpvdD* or *ΔcoaB* + Pf4 or *ΔpqsA* + Pf4 or PAK + Pf1: p ≤ 0.01; PAK: p>0.05. All other bars: p≤0.001. Comparison of PAO1 vs. its mutants: *ΔlasR/rhlR*: p≤0.05; *ΔpvdD*: p ≤ 0.001; All other bars: p>0.05. Comparison PAO1 vs PAK: p≤0.01.

**Fig 5:**
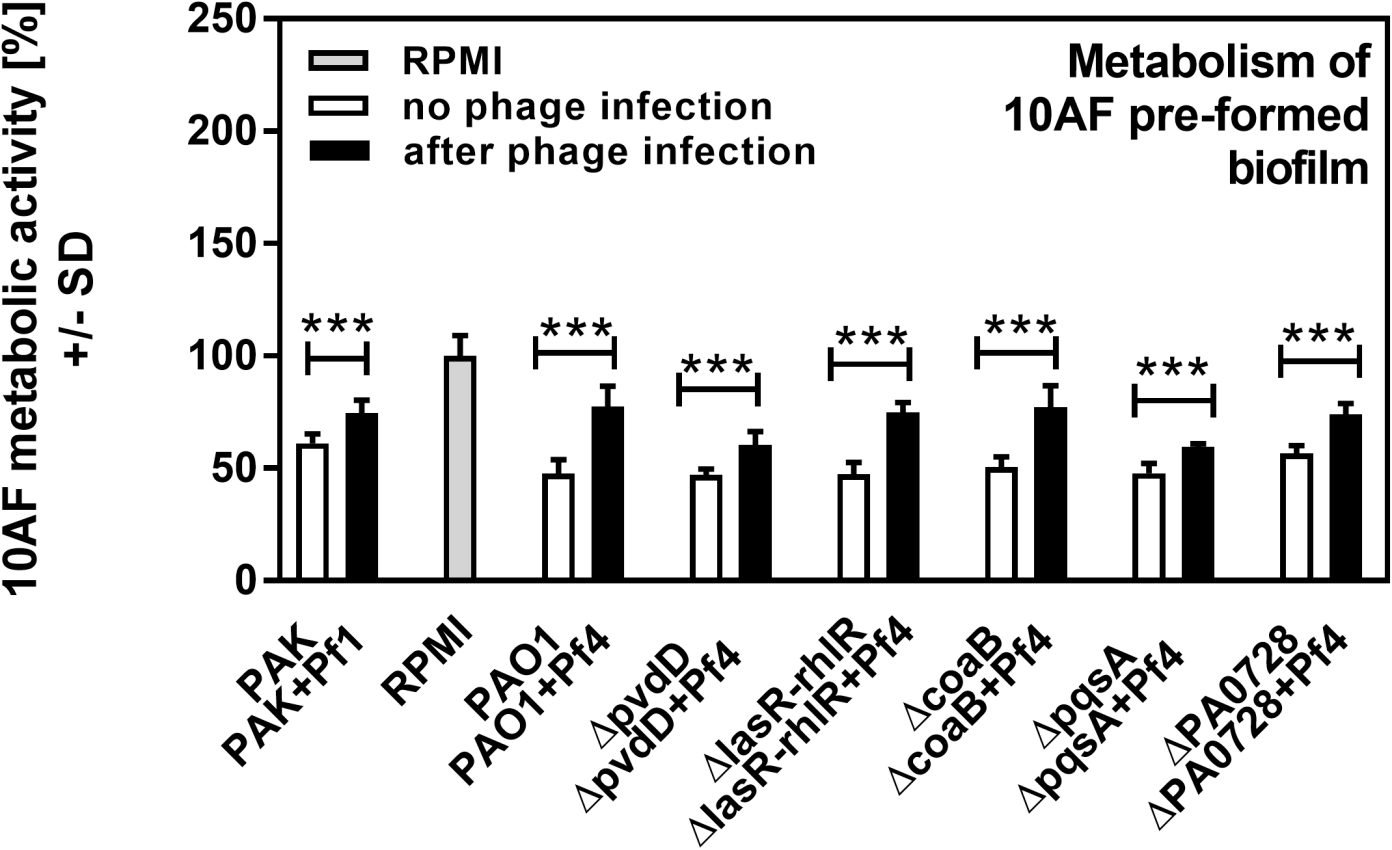
Effect of phage infection on preformed Af biofilm, as studied in liquid in wells. Black bars show inhibition of Af biofilm by planktonic supernatants of Pa after acute infection with phage, white bars show inhibition of Af biofilm by planktonic supernatants of the same strains of Pa cultured in the absence of phage. Grey bar shows negative control (RPMI medium only). Horizontal asterisks compare the bars at the ends of the horizontal brackets; three asterisks represent differences of p ≤ 0.001, respectively, comparing each Pa isolate uninfected vs. after phage infection. Comparison of all vs RPMI (the Af control, grey bar): All bars: p≤0.001. Comparison PAO1 vs. its mutants: *ΔPAO728*: p≤0.001. All other bars: p>0.05. Comparison PAO1 vs PAK: p≤0.001.

Pa secretes a variety of antimicrobial compounds [5,6,7]. To investigate whether Pf phage affected antimicrobial production, Pa cultures were infected with purified Pf phage (10^7^ PFU/ml). After overnight growth, Pf phage titers were approximately 10^10^ PFU/ml (**Fig 1A**), comparable to those achieved by Pa biofilms [11,12,13]. Filamentous Inovirus bacteriophage, such as fd, M13, and Pf, typically do not lyse their bacterial hosts, but are rather continuously extruded from them[17]. Consistently, bacterial densities were comparable between Pa cultures not infected with Pf phage and cultures infected with Pf phage (**Fig 1B**). To determine if infection by Pf phage affected antimicrobial production, Af biofilms (either preformed or forming) were exposed to filter sterilized supernatants and Af metabolic activity was measured using the XTT assay. The antifungal properties of supernatants collected from Pa infected with Pf phage, against both Af forming biofilms or preformed Af biofilm, were reduced to levels comparable to growth media alone (**Figs 2–5**). This reduction in Af metabolic activity was observed in two strains of Pa infected with different strains of Pf phage (PAO1 and PAK infected with Pf4 or Pf1, respectively). These results suggest that antifungal production by Pa is reduced by acute infection by Pf phage.

In wild type Pa (strain PAO1), the Pf phage is integrated into the bacterial chromosome as a prophage [16]. Thus, it is possible that wild type Pa could produce basal levels of phage not detected by the plaque forming assays we utilized to quantify Pf phage (lower limit of detection, 2000 PFU/ml), and this basal level of Pf phage production could affect results. To address this possibility, Pa strains unable to produce Pf phage—the phage integrase mutant *ΔPA0728* [11] and the major coat protein mutant *ΔcoaB* (S1 **Fig**)—were used. Both Pf phage-deficient mutants produced supernatants with antifungal properties comparable to wild type supernatants (**Figs 2–5**), indicating that low-level production of Pf phage by wild type bacteria (<2000 PFU/ml) were not influencing the antifungal properties of Pa supernatants. The phage-deficient mutants *ΔPA0728* and *ΔcoaB* are susceptible to infection by exogenous Pf phage (**Fig 1B and S1 Fig**). When *ΔPA0728* and *ΔcoaB* were infected with purified Pf phage, the antifungal properties of their supernatants were reduced to levels similar to those of wild type bacteria infected with Pf phage (**Figs 2–5**). Because our previous studies indicate that purified Pf phage act to *decrease* the metabolic activity of Af biofilms [15], our results here raise an additional possibility that Pf phage infection results in Pa supernatants in which antimicrobials produced by Pa are decreased by infection with Pf phage.

Pa produces many antimicrobial compounds, many of which are regulated by quorum sensing–a system used by bacteria to coordinate gene expression in response to changes in bacterial population density. There are three known quorum systems in Pa—las, rhl, and pqs. To determine if quorum-regulated antimicrobial production was affected by phage infection, we tested supernatants collected from Pa where the las, rhl, and pqs quorum systems were genetically disabled. Under the conditions tested, disabling the las/rhl quorum systems significantly reduced the antifungal properties of Pa supernatants against both forming (**Fig 2**; p <0.01) and preformed (**Fig 4**; p <0.05) Af biofilms (**Figs 2–5**). When the pqs quorum system was disabled (*ΔpqsA*), however, the antifungal properties of Pa supernatants not infected with Pf phage were not significantly reduced against forming or pre-formed Af biofilms (**Figs 2–5**). When these quorum-deficient strains were infected with Pf phage, supernatants collected from both *ΔlasR/rhlR* and *ΔpqsA* exhibited impaired antifungal activities against both forming and pre-formed Af biofilms (**Figs 2–5**). Collectively, these results suggest that Pf phage infection reduces las/rhl-dependent and -independent antimicrobial production in Pa.

The las/rhl quorum system regulates several antimicrobials, including the virulence factor pyoverdine, a high-affinity iron chelator [18]. Because iron chelators can inhibit Af biofilms [19] and studies with a variety of Pa mutants have pinpointed pyoverdine as the key fungal inhibitor ([20] and G. Sass *et al*., submitted for publication), we hypothesized that Pf phage infection would reduce pyoverdine biosynthesis, reducing the antifungal properties of Pa supernatants. To test this hypothesis, the pyoverdine biosynthesis gene *pvdD* was inactivated. The antifungal properties of *ΔpvdD* supernatants against forming and pre-formed Af biofilms were significantly (p <0.001) reduced compared to the parent (**Figs 2–4**), confirming that pyoverdine contributes significantly to the antifungal properties of Pa supernatants. The addition of Pf phage to the *ΔpvdD* strain, however, further reduced the antifungal properties of *ΔpvdD* supernatants to levels comparable to those observed with fresh growth medium (**Figs 2–5**).

**Fig 6:**
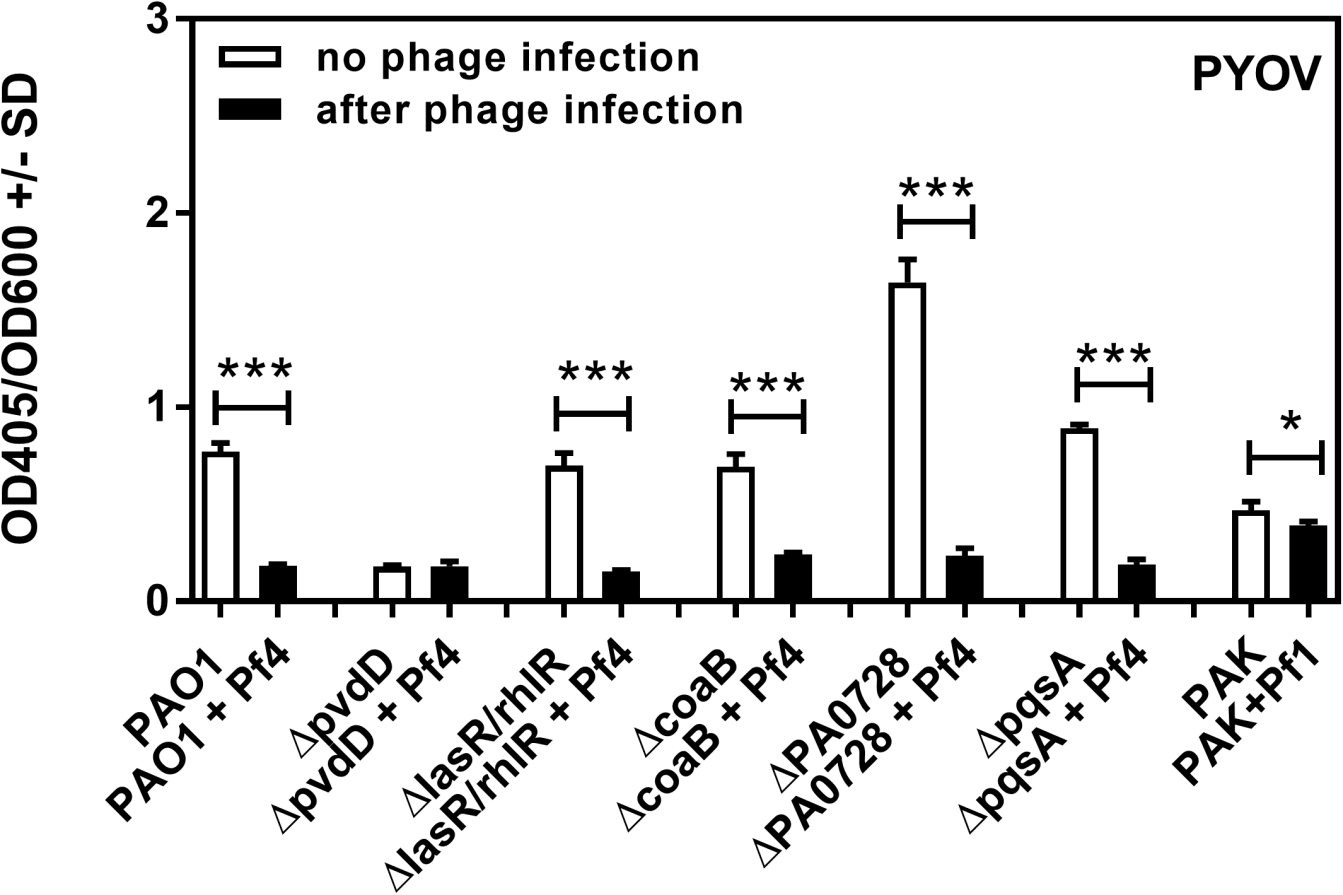
Effect of phage infection on the production of pyoverdine by Pa. Black bars show pyoverdine production after phage infection, white bars show basal pyoverdine production by planktonic supernatants of the same strains of Pa not infected by phage. One and three asterisks make comparisons within each pair framed by the horizontal bracket; p ≤ 0.05, p ≤ 0.001, respectively.

To determine if Pf phage production inhibited pyoverdine biosynthesis, we measured pyoverdine in Pa supernatants. When not infected by Pf phage, pyoverdine levels were reduced to negligible levels in the *pvdD* mutant relative to wild type bacteria (**Fig 6**). Infection by Pf phage reduced pyoverdine levels in all strains tested to levels similar to those observed for *pvdD* (**Fig 6**), especially the phage-deficient mutant *ΔPA0728*. Collectively, these results indicate that infection by Pf phage inhibits pyoverdine production by Pa, and that pyoverdine is the major inhibitor of Af produced by the uninfected Pa.

## Discussion

Pa and Af are the most prevalent bacterial and fungal pathogens isolated from CF airways, respectively, and clinical CF isolates of these pathogens often resist antibiotic treatment [21,22]. Recently, there has been renewed interest in the use of bacteriophages [23] and mycoviruses [24] as alternatives to antibiotics to treat microbial infections. However, the consequences of using viruses as therapeutics are not well understood and the impact viral infection has on intermicrobial interactions—such as those between Pa and Af—remain unclear. Understanding how viruses affect intermicrobial interactions may facilitate the development of novel therapeutic approaches and improve responses to current therapies.

In this study, we measured the impact acute Pf phage infection had on the antifungal properties of Pa supernatants. Through a combination of biochemical and genetic approaches, we determined that Pf phage infection strongly inhibits the antimicrobial activity of Pa supernatants against Af biofilms. Our studies suggest that Pf phage infection significantly reduced both quorum-regulated and quorum-independent antimicrobial production. Previous work, however, suggests that chronic phage predation results in an increase in the expression of virulence-associated genes in Pa [25], a finding that directly opposes our results. That study, in contrast to our work, investigated Pa variants that were resistant to infection by phages PP7 and E79, not bacteria actively producing phage. Thus, the use of different species of phage in addition to examining bacteria not actively infected by phage, or differences between acute and chronic effects of phage predation, may explain the discrepancy between the two studies.

Whereas our study links acute Pf phage infection with reduced antimicrobial production by Pa, our study had some limitations. For example, Inoviruses like Pf phage typically do not lyse and kill their bacterial hosts [17]. Thus, Pf phage may not be suitable for therapeutic use. However, superinfective forms of Pf phage that are capable of causing bacterial lysis can be produced by *P*. *aeruginosa* biofilms [26], although the mechanism by which superinfective Pf phage cause bacterial lysis is currently poorly understood. We also used planktonic Pa cultures infected with purified Pf phage. It is possible that supernatants collected from bacteria growing as a biofilm that are spontaneously producing Pf phage would produce different results. Additionally, growing Pa and Af in co-culture would likely affect antimicrobial production by Pa, as would the use of different species of bacteriophage. Studies investigating these ideas are currently underway. We chose to monitor the antifungal properties of Pa supernatants by the XTT assay of metabolism, which measures the reduction of the dye XTT. While XTT is often used to assay Af viability and antifungal susceptibility [27,28,29], this assay has limitations. For example, our XTT results do not rule out the possibility that Af switched to non-oxidative, fermentative metabolism. Traditional methodologies such as CFU counting could not be used to enumerate viable Af because Af is a multinucleate filamentous microorganism, and has perforate intercellular septa, CFU counting is inaccurate, and that may be especially problematic in Af biofilms [30,31].

A key finding of our study was that the production of the virulence factor pyoverdine, a high-affinity iron chelator that is important for virulence in Pa [20,32], was severely inhibited in Pa cultures infected by Pf phage. This observation is consistent with previous studies analyzing gene expression in Pa. For example, in clinical Pa isolates, Pf phage gene expression was upregulated while pyoverdine biosynthesis genes were downregulated [33]. Similarly, when Pa was cultured in semi-viscous environments—conditions that are likely to be encountered at sites of infection—Pf phage genes were upregulated and pyoverdine biosynthesis genes were downregulated [34]. These observations suggest that Pf phage and pyoverdine biosynthesis are inversely regulated in Pa.

It was interesting that the phage-deficient mutant *ΔPA0728* produced far more pyoverdine than the *ΔcoaB* mutant in the absence of exogenous Pf phage, even though both are phage-deficient. One explanation involves the double stranded DNA replicative form (RF) of the Pf phage. During lytic replication, the RF serves as a template for the transcription of phage genes and the *ΔPA0728* mutant cannot maintain the RF [35]. Thus, the expression of all phage genes would be reduced, which may affect other bacterial functions (i.e., pyoverdine biosynthesis). The *ΔcoaB* strain, however, may still produce low levels of the RF in the cytoplasm, but because the major coat protein CoaB is not transcribed, mature phage particles are not produced. However, other genes present on the RF—genes that may be interfering with pyoverdine biosynthesis–could still be produced in the *ΔcoaB* background.

Pf phage infection completely abolished the antifungal properties of Pa supernatants in two different assays (BCAM and BHAM) and the inhibition of pyoverdine biosynthesis explains most, but not all of this observation. Thus, other molecules with antimicrobial activity—both quorum-dependent and -independent—are likely affected by Pf phage infection. One possible explanation is the increased metabolic burden acute phage infection imposes on Pa–a large metabolic burden might be expected to reduce the production of antimicrobials in general. Another possibility involves global regulatory systems in Pa. For example, MvaT and MvaU are global transcriptional regulators in Pa that regulate both Pf phage replication and virulence factor production [35,36]. Activation of such global regulatory mechanisms by acute Pf phage infection may explain the decreased antimicrobial properties of Pa supernatants. Further work will be required to determine if this is true.

Overall, our data indicate that acute predation by Pf phage reduces the antifungal properties of Pa against Af biofilms. At sites of infection, the reduced antifungal properties of Pa infected with Pf phage may establish conditions that allow co-infection with Af, increasing disease severity. Furthermore, while it is not yet clear if our findings are applicable to other species of bacteriophage, our results raise the possibility that the use of some bacteriophage as therapeutics may promote colonization with other pathogens that could increase disease severity.

## Materials and methods

### Bacterial strains and culture conditions

All strains, plasmids, and PCR primers are listed in **Table 1**. The indicated Pa strains were grown in RPMI-1640 (Lonza) at 37°C with shaking unless otherwise stated. Af was cultured as previously described [37] and is elaborated below.

**Table 1:** Bacterial strains, plasmids, and PCR primers used in this study.

Strain or plasmid	Genotype, description, or sequence	Source
*P*. *aeruginosa*		
PAO1	Wild type	[38]
PAK	Wild type	ATCC 25102
*ΔcoaB* *ΔPA0728*	PAO1 Δ*PA0723* PAO1 Δ*PA0728*	This study [11]
*A*. *fumigatus*		
10AF	A virulent Af strain	[39]
Bacteriophage		
Pf4	Isolated from PAO1 biofilm	[11]
Pf1	Pf phage that infects PAK	ATCC 25102-B1
Plasmids		
pDONRpEX18Gm	pEX18Gm with Gateway donor site	[40]
pDONRpEX18Gm::*ΔPA0723*	Deletion construct targeting *coaB* (*PA0723*)	This study
Primers		
PA0723F01-GWB1	GGGGACAAGTTTGTACAAAAAAGCAGGCTCCCAGGACAAACAAGACAAGAC	This study
PA0723R01	CACCCGTTACGCCTTGCGCGATGCGTTGCTTCATTGCTTTC	This study
PA0723F01	GCGCAAGGCGTAACGGGTG	This study
PA0723R01-GWB2	GGGGACCACTTTGTACAAGAAAGCTGGGTACAGTTCGCCTTCCTTGCATTC	This study
PA0723 seq-F	CCAGGACAAACAAGACAAGAC	This study
PA0723 seq-R	CACACCGACCAGAGCACC	This study
M13F(-21)	TGTAAAACGACGGCCAGT	
M13R	CAGGAAACAGCTATGAC	
Pf4F	AGCAGCGCGATGAAGCAAT	[12]
Pf4R	TAGAGGCCATTTGTGACTGGA	[12]

### Phage purification

Pf phage were purified from bacterial supernatants by precipitation with polyethylene glycol (PEG8000) followed by dialysis against PBS, as previously described [11].

### Preparation of Pa supernatants

Frozen stocks of Pa (either PAO1 or PAK) were streaked onto LB agar plates. After overnight growth at 37°C, an isolated colony was selected and grown to an optical density (OD_600_, Genesys 20 spectrophotometer, Thermo) of ~0.1 in 5 ml RPMI-1640 at 37°C with shaking. At this time, either 100 μl PBS or 100 μl purified Pf4 or Pf1 (both at 10^7^ phage per ml) were added. After overnight growth, bacterial densities were measured by absorbance (OD_600_). Bacteria were then pelleted by centrifugation (9,000xg for 10 minutes) and the supernatants were filter sterilized using syringe filters with 0.2 μm pores (Millipore). Bacteriophage titers were estimated by enumerating PFU/ml as described [35].

### Construction of the *coaB* mutant

The *ΔcoaB* strain was created by allelic exchange [40]. Briefly, the *ΔcoaB* mutant allele was created by using two sets of PCR primers (PA0723F01-GWB1 and PA0723R01; PA0723F01 and PA0723R01-GWB2) targeting the upstream and downstream regions of *coaB* (*PA0723*). These PCR products were gel purified (Qiagen) and subsequently joined via splicing by overlap extension (SOE) PCR to generate an in-frame deletion allele with flanking attB1 and attB2 sequences. The *attB*-tailed SOE PCR product was recombined with pDONRpEX18Gm using BP Clonase II (Invitrogen), producing the allelic exchange vector pDONRpEX18Gm::*ΔPA0723*. The deletion allele was confirmed by sequencing using M13F(-21) and M13R primers. To generate *coaB* deletions in the Pa chromosome, pDONRpEX18Gm::*ΔPA0723* was transformed into the donor strain *Escherichia coli* S17.1 (selected on agar containing 10 μg/ml gentamicin) and then introduced into Pa via biparental mating [40]. Merodiploids were selected on VBMM agar containing 100 μg/ml gentamicin and subsequently streaked on no-salt LB agar containing 15% sucrose for counter-selection. Gentamicin-sensitive, sucrose-resistant strains with the mutant alleles were identified by PCR and confirmed by sequencing using the primers PA0723 seq-F and PA0723 seq-R.

Pf phage production by the *coaB* mutant was measured by PCR using primers targeting the re-circularization sequence in the Pf genome [12], as described with modifications. Briefly, supernatants from PAO1, *ΔcoaB*, or *ΔcoaB* infected with Pf4 (*ΔcoaB*+Pf4) were prepared as described above. One-milliliter aliquots of these supernatants were then boiled for 10 minutes to denature any intact phage particles, releasing the circular DNA Pf phage chromosome. One μl of boiled supernatant was used as a PCR template using primers Pf4F and Pf4R. The presence of Pf4 in filtered bacterial supernatants was confirmed by the amplification of an 839-bp region corresponding to the re-circularization region of the Pf4 chromosome.

### Assays used for the determination of Pseudomonas effects on Aspergillus

Effects of bacterial supernatants on Af biofilm formation and preformed Af biofilm were studied in wells as described previously [19,37], or in agar-based assays, as described below. To combine results obtained in concurrent assays on separate plates, test results from each plate were converted to % of the RPMI control (= 100%) prior to combining results.

### Forming (BCAM: Bioassay-Conidia-Agar-Metabolic) and preformed (BHAM: Bioassay-Hyphae-Agar-Metabolic) 10AF biofilm plate assays on agar

Bacto agar (1.25 g) (BD Biosciences, Durham, NC) was added to 25 ml distilled water and autoclaved. After cooling to 56°C, 75 ml of RPMI 1640 medium (Lonza, Walkersville, MD) and 2.5x10^4^ Af (strain 10AF, [39]) conidia/ml agar were added. The conidia-containing agar was distributed into the inner 60 wells of sterile flat bottom 96 well cell culture plates (COSTAR, Corning, NY) at 100 μl/well. Upon agar solidification, plates were either loaded immediately with supernatants or control (= BCAM assays) or incubated at 37°C for 24 hours before loading (= BHAM assays). Immediately before loading, all empty peripheral wells of each 96 well plate were filled with 200 μl of sterile water to limit evaporation from the test wells. Test wells were loaded with 100 μl supernatants collected from the indicated strain of Pa. Control wells on each test plate contained 100 μl of RPMI 1640 medium, allowing test results to be normalized between plates. Loaded plates were incubated at 37°C for 24 hours. On the agar, hyphal mats of biofilm form, as verified by visual microscopy, showing the same arrangement as has been studied in liquid wells, studied by visual, confocal and electron microscopy [41].

### XTT metabolic assay

Plates were evaluated by XTT metabolic assay as described [37]. Briefly, 100 μl of an XTT-menadione mixture [150 μg/ml XTT and 30 μM menadione] were added to each test well and incubated at 37°C for 30 minutes (BCAM assays) to 1 hour (BHAM assays). Supernatant from each well (100 μl) were assayed using a plate reader at 490 nm (Opsys MR, DYNEX Technologies, Chantilly, VA).

### Pyoverdine measurement

Pyoverdine production was measured in bacterial supernatants at an optical density of 405 nm [42]. Relative PYOV expression was calculated by normalizing pyoverdine measurements (OD_405_) to bacterial growth (OD_600_) using the formula: OD_405_ / OD_600_.

### Statistical analysis

Statistical analysis was performed using Prism GraphPad software; means with SD were calculated and plotted unless specified otherwise. An unpaired t test was used to calculate p values. P ≤0.05 were considered significant.

## Supporting information

S1 Fig Characterization of Pf phage production in the *coaB* mutant. The presence of Pf phage (strain Pf4) in the supernatants of the indicated bacterial strains was detected by PCR. Primers targeting the re-circularization region of the Pf4 genome were used [12] to detect Pf phage in supernatants of the indicated strains. These primers can only amplify the circular Pf DNA chromosome (producing an 839 bp product) and not the linear prophage integrated into the Pa chromosome.
(TIFF)
