# Supplementary figures and images for: Effect of acute predation with bacteriophage on intermicrobial aggression by Pseudomonas aeruginosa
Source: PLoS One. 2017 Jun 16;12(6):e0179659. doi: 10.1371/journal.pone.0179659 (PMC5473581; doi:10.1371/journal.pone.0179659)

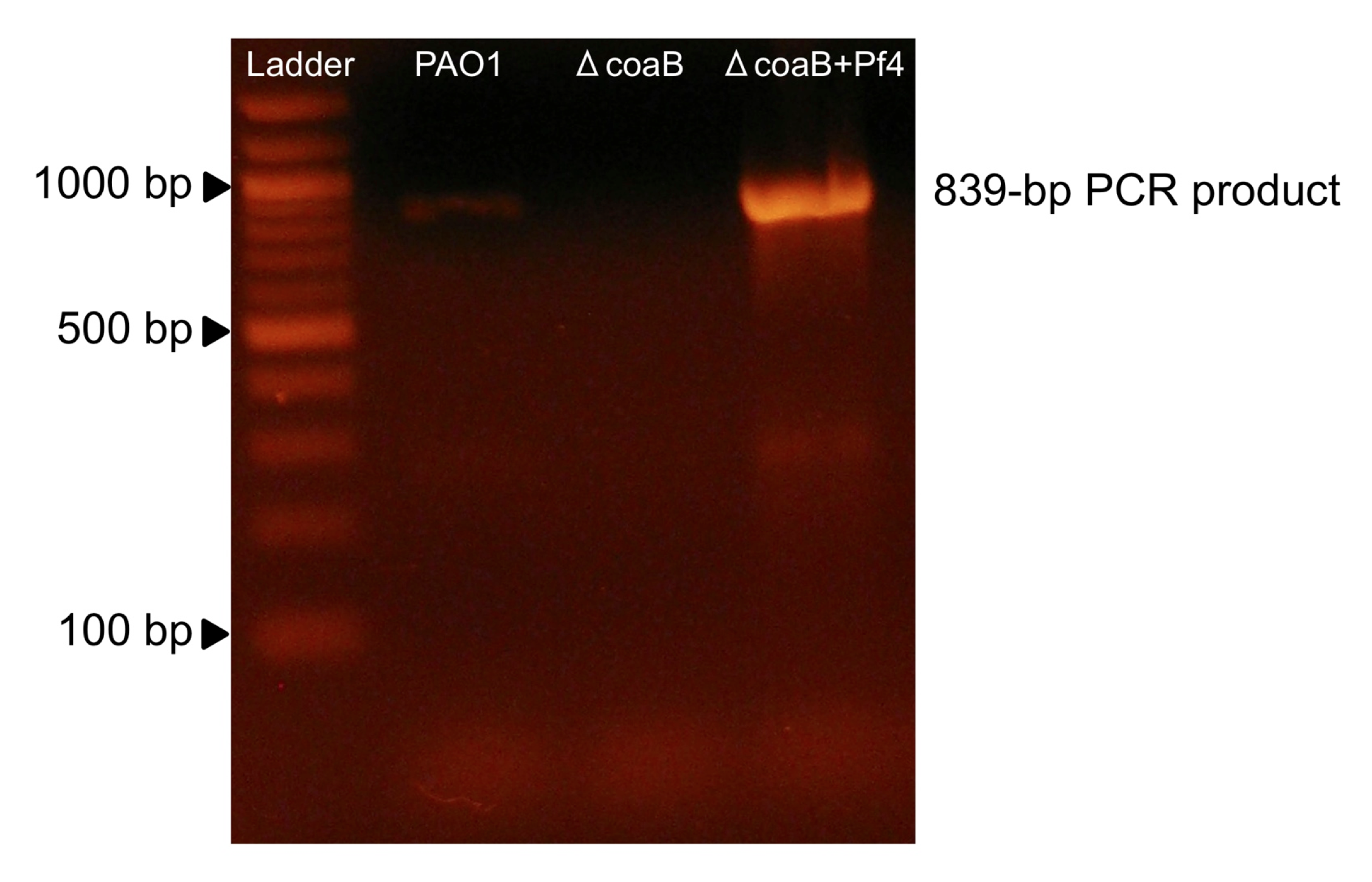

Supplement: S1 Fig — The presence of Pf phage (strain Pf4) in the supernatants of the indicated bacterial strains was detected by PCR. Primers targeting the re-circularization region of the Pf4 genome were used [12] to detect Pf phage in supernatants of the indicated strains. These primers can only amplify the circular Pf DNA chromosome (producing an 839 bp product) and not the linear prophage integrated into the Pa chromosome. (TIFF) [file pone.0179659.s001.tiff]
